# The Muscular Factor in Cases of Delay in Walking

**Published:** 1909-03-20

**Authors:** 


### Diseases of Children.

### THE MUSCULAR FACTOR IN CASES OF DELAY IN WALKING.

A child normally learns to walk between a year and 18 months of age. As soon as the infant has developed the idea of assuming the upright position by holding on to some suitable support, it begins to make steps whilst clinging to that support. The uncertainty of those steps is at first comparable to an extreme degree of ataxy. The feet may even be put down upon the ground the wrong way round, and steps that are too long lead to stumbles and falls. This uncertainty gradually disappears in from four to six weeks, and then the second stage of learning to walk begins; the supports are let go, and the infant tries to balance its own body without external aid. Swayings of the trunk, wavings of the arms as a sort of balancing-pole, are precisely similar to those of the adult who is learning to skate; they last two or three weeks, and then as a rule disappear.

There are considerable variations in the times at which children who cannot be called abnormal begin to walk, and there are also wide differences in the time that elapses between the first attempts and the final ability to walk unaided. The infant's intelligence here plays a considerable part. Moreover, the actual painfulness of accidents may be a factor in the delay that sometimes occurs in so far that the child is unwilling to repeat attempts that have had untoward effects in the immediate past.

It is very hard to draw any hard and fast line between physiological and pathological departures from the normal as regards lateness in learning to walk. The causes of abnormal lateness in walking may be broadly summarised thus:

1. *General Disorders of Development.*—(*a*) Congenital or hereditary—such as premature birth. (*b*) Acquired—such as marasmus from ill-feeding, or from illness.

2. *Local Disorders.*—(*a*) Affections of the nervous system. (*b*) Affections of the supporting tissues.

General disorders of development vary in their action according to their degree of intensity. Upon the whole they are not so potent in delaying the ability of the child to walk as might be expected. Infants that survive premature birth usually learn to walk at approximately the right time. Temporary illnesses or disorders of metabolism may be recovered from so quickly that little or no mark of them is left behind. Severe marasmus, however, may be followed by great delay in the development of the infant in every respect, including the power of walking. Lateness in walking is here, however, but one symptom amongst many others and it requires no particular treatment itself.

It is otherwise, however, with the second of the groups mentioned above, which includes most of the cases in which lateness in walking brings the patient to the doctor.

Amongst nervous diseases may be mentioned, first of all, impairment of mental capacity, of which there are all degrees. In all such cases walking may be much delayed. Only one variety is open to cure, and that is cretinism. It is often worth while exhibiting thyroid extract in cases which may at first seem hopeless, in the hope that cretinism may be at the root of the pathology of the case. Other diseases of the nervous system that may be mentioned are spina bifida, spina bifida occulta, hæmatomyelia after whooping cough, acute anterior poliomyelitis, transverse myelitis, congenital progressive muscular atrophies, infantile diplegia, and, although rather a muscular than a nerve disease, one or other of the primary muscular dystrophies. The diagnosis in each of these cases will usually be fairly clear if the reflexes and the electrical reactions are taken into account.

Of changes in the supporting apparatus, rickets is the disease to which most cases of "simple" late walking are attributed; it is worth while to consider what relation rickets really has to late walking. Mothers dislike being told their children are ricketty. Is it possible that some of the late walking commonly attributed to rickets may really be due to something else? Forest suggests that this is so, and his view is at any rate of interest.

In the first place many a ricketty child shows no delay in walking whatever, with the result that bandy legs, genu valgum, or contracted pelvis follow. Moreover, there are numerous children in their second year of life who are almost or quite free from the accepted symptoms of rickets and yet exhibit a greater or less degree of muscular flaccidity, especially in their lower limbs. It is true that they can move their legs about when they are sitting or lying in bed, but when a limb is passively raised and then let go it falls as though its muscles were lacking in tone. When the child is held up in the standing posture and urged to walk the legs remain passive as though the infant had no power to stiffen them. The knee-jerk may be present and often quite brisk, unless the child is ill from some intercurrent malady, in which case the knee-jerk is often very difficult to elicit—a point which is true of most children when they are ill. There is no definite alteration in the electrical reactions of the muscles, although it may be difficult to elicit any contraction at all with the maximum current that the infant can bear. One must allow either that idiopathic muscular weakness is a manifestation of rickets or else that lateness of walking due to this condition of the muscles is not so often ricketty as is generally stated. In either case the other usual signs of rickets are not infrequently quite absent, and there may have been no error in the patient's dietary at all.

As regards the histological changes in the muscles little can be said, for the condition is not by itself fatal. In cases that have died of an intercurrent malady, however, the following histological findings have been recorded: the muscle fibres exhibit deficiency in their transverse striation, increased clearness of their longitudinal striation, considerable proliferation of their nuclei, reduction in the breadth of the individual fibres, and increase in the interstitial fat and connective tissue.

A name suggested for the condition is *myatonia congenita* in preference to rickets. Most cases are fortunately mild, so that patience is all that is required for natural cure. In other instances, however, the delay in walking may be alarming to the parents. When it is realised that the cause of the delay is in the muscles rather than in the bones it is clear that any treatment undertaken is most effective when it takes the form of massage and the gentle application of electricity.

